# Education and Training in Clinical Neuropsychology: Recent Developments and Documents From the Clinical Neuropsychology Synarchy

**DOI:** 10.1093/arclin/acy075

**Published:** 2018-10-11

**Authors:** Glenn Smith, CNS

**Affiliations:** Department of Clinical and Health Psychology, University of Florida, Gainesville, FL, USA

**Keywords:** Clinical neuropsychology, education, training, competencies

## Abstract

The Clinical Neuropsychology Synarchy (CNS) interfaces with the American Psychological Association and affiliated organizations to address issues and advances in specialty training. The past several years have seen the development and dissemination of several initiatives pertinent to specialty training. Among these initiatives was the creation of a taxonomy for education and training in clinical neuropsychology. In additional there has been a movement towards competency-based education that has become codified in the APA’s new Standards for Accreditation. Calls for competency-based education have also influenced the expectations of the APA’s Committee on Recognition of Specialties and Proficiencies in Professional Psychology. As the convener of national clinical neuropsychology organizations the CNS has overseen the development of relevant documents for our specialty. This paper presents three documents critical to training in our field that were developed through the CNS and approved by its member organizations. The first is the Taxonomy for Education and Training in Clinical Neuropsychology. The second is Entry Level Competencies for Clinical Neuropsychology and the third is a distillation of the entry-level competency document for the purpose of identifying competencies to be addressed at the post-doctoral residency level.

## Introduction

**Fig. 1.**
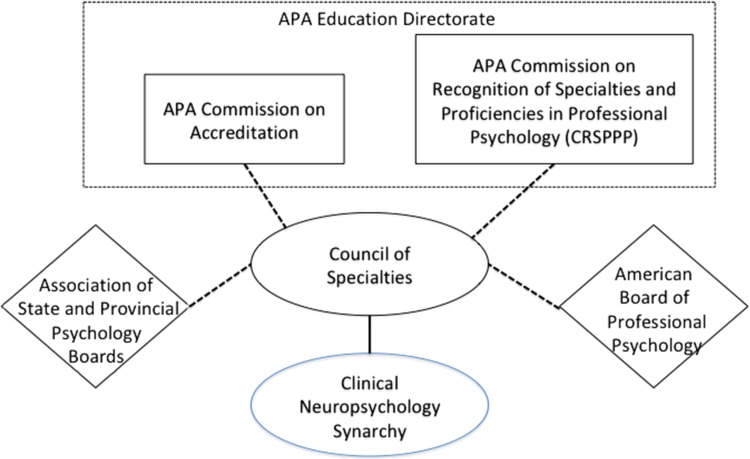
Reporting relationships among entities involved in specialty training guidelines.

In the past 6 years the Education Directorate of the American Psychological Association (APA) has been very active in seeking to standardize nomenclature and practices as they pertain to education and training in professional psychology specialties. The APA Education Directorate has taken advantage of the infrastructure that exists (technically outside of the APA) to reach out to the specialties to both seek input and disseminate “standards” to the specialties. Fig. 1 depicts that infrastructure including the place of the Clinical Neuropsychology Synarchy (CNS) within this infrastructure. The initiatives that have emanated from APA’s Education Directorate have resulted in three key documents critical to training in our field that were developed through the CNS and approved by its member organizations. The first is the Taxonomy for Education and Training in Clinical Neuropsychology. The second is Entry Level Competencies for Clinical Neuropsychology and the third is a distillation of the entry-level competency document for the purpose of identifying competencies to be addressed at the post-doctoral residency level. Presented below are the roles played by various entities presented in Fig. 1 followed by the text of the three key documents.

## The role of APA Commission on Recognition of Specialties and Proficiencies in Professional Psychology (CRSPPP)

As suggested by its name, CRSPPP is the organization within the APA that formally recognizes professional psychology specialties. There are currently 15 specialties recognized by CRSPPP. In 2012, CRSPPP published *Education and Training Guidelines: A Taxonomy for Education and Training in Professional Psychology Health Service Specialties* (APA, 2012). This document was the culmination of efforts by CRSPPP, the Council of Specialties (CoS), the American Board of Professional Psychology (ABPP), the APA Commission on Accreditation (CoA) and others to address the problem reflected in the document’s statement of need:

“There has been a growing need for a taxonomy to guide those seeking education and training in a recognized specialty.^1^ Such a taxonomy would also provide a structure for the field in general to facilitate better communication concerning how professional psychologists are educated and trained beyond the broad and general training consistent with accreditation standards (APA, 2009b). Currently there is not consistent use of definitions or terms in the education and training community to describe training opportunities in areas recognized as specialties by APA (APA, 2009a, Section 90-5). For example, in describing training opportunities, education and training departments and programs use a range of terms like *area of study*, *track*, or *concentration*. These terms may refer to the same or to different amounts of course work or practica from department to department or from program to program. Such inconsistency jeopardizes a consistent “truth in advertising” that would be helpful to students as they seek graduate education or to later describe the educational and training opportunities provided in their graduate or postgraduate education.”

“Lack of consistency in the use of such terms is also evident across professional organizations and groups whose focus is on education and training in professional psychology. For example, the Association of Psychology Postdoctoral and Internship Centers (APPIC) asks programs to describe the types of training available using the terms major rotation and informal/minor/external rotation, whereas the Committee on Accreditation (CoA) uses the phrase areas of emphasis to refer to training opportunities in specialty areas other than the substantive traditional practice areas of clinical, counseling, and school (APA, 2003, IR C-6[a]).” (APA, 2012, p. 4.)

Having laid out a set of common terms and constructs for the taxonomy CRSPPP charged the CoS to obtain from the specialties, definitions of the terms within the taxonomy as they pertained to training in given specialty.

In addition, documentation of the entry-level competencies of that specialty is expected to be part of petitions to CRSPPP for specialty recognition or renewal for each specialty. In anticipation of pursuing specialty recognition renewal in 2017 is was clear that the Houston Conference document (Hannay et al., 1998) needed supplementation to include an enumeration of competencies that aligned with recent competency standards in professional psychology (Kaslow, 2004). Thus a statement of entry level entry-level competencies for clinical neuropsychology was developed, vetted and approved through CNS.

## The Role of the APA Commission on Accreditation

In 2015, the Commission on Accreditation’s new Standards of Accreditation (SoA; APA, 2015) were approved by the APA Council of Representatives. These standards went into effect for programs being reviewed after January 7, 2017. These standards have been a major impetus for defining and training to competencies in health services psychology. The competency documents provided below in this manuscript represent our specialty’s efforts to align with this trend. Of specific import to specialty training, and relevant especially at the post-doctoral residency level, the SoA describes advanced competency levels as follows:

- Level 1: Advanced competencies fundamental to health service psychology
- Level 2: Competencies relevant to the program’s aims or area of focus
- Level 3: Competencies that are consistent with a designated specialty

Subsequent to issuing the new standards of accreditation the CoA has seemed to increase its attention to accreditation of post-doctoral training programs. In order to proceed with this the CoA has begun to consider the competencies that should be addressed in post-doctoral training. Thus in the spring of 2017 the CoA, through the CoS, asked the specialties delineate competencies to be consider during the review of post-doctoral training programs seeking specialty accreditation.

## The Role of the Council of Specialties

Specialties in professional psychology recognized CRSPPP are expected to have a specialty council. In fact, the first criterion in a petition for specialty recognition is

“Criterion I. Administrative Organizations. The proposed specialty is represented by a specialty council of one or more organizations that provide systems and structures sufficient to support the organized development of the specialty.” (APA, 2011, p. 3)

CoS is not an official APA committee. CoS does include a liaison from CRSPPP as well as liaisons from other groups such as Association of State and Provincial Boards of Psychology, the American Board of Professional Psychology and the Association of Psychology Graduate Students. CoS advocates for specialty issues within the APA structure through CRSPPP. Similarly when APA encounters issues germane to more than one specialty it will work with and through CoS to address these cross-specialty issues. Each of the 15 specialty councils sends a representative to the Council of Specialties (www.cospp.org). Generally that representative is the chair of the specialty council.

## The Role of the Clinical Neuropsychology Synarchy

Aside from the broader Clinical, School, and Counseling areas, clinical neuropsychology is the longest recognized specialty in psychology. As such, it maintains one of the most experienced specialty councils. Our council is named the Clinical Neuropsychology Synarchy (synarchy means to govern together and is the antonym of anarchy). Our specialty council retains this old name in recognition of the clinical neuropsychologists who did the heavy lifting that resulted in the recognition of neuropsychology as a specialty in the first place.

The CNS is made up of representatives of the major American clinical neuropsychology organizations. This includes organizations that represent individual clinical neuropsychologists and organizations that represent institutions that are training or credentialing the next generation of clinical neuropsychologists. The member organizations of CNS are listed in Table 1.

**Table 1.** CNS membership during the development of the documents described herein

These organizations can be organized as follows:		
*Organizations representing individual neuropsychologists*	*Organizations representing training institutions*	*Organizations engaged in certification*
Society of Clinical Neuropsychology	Association of Post-Doctoral Programs in Clinical Neuropsychology	American Board of Clinical Neuropsychology
National Academy of Neuropsychology	Association of Internship Training in Clinical Neuropsychology	American Board of Professional Neuropsychology*
American Academy of Clinical Neuropsychology	Association of Doctoral Education in Clinical Neuropsychology	
Association of Neuropsychology Students*		

*Liaison.

It is through the structure depicted in Fig. 1 that the organizations represented in Table 1 responded to APA calls for a specialty-specific education and training standards and documents. In the case of each of three documents presented herein, the CNS Chair (GES) empaneled subcommittees from CNS membership to respond to requests from CoS. In each case representatives from organizations with a major stake in the document (e.g. ADECN, AITCN, and APPCN for the taxonomy document) volunteered to draft preliminary documents. These documents were then circulated via CNS to all its members for comment and suggested revision. In each case the CNS subcommittee collated and responded to suggested revisions before returning the document to CNS members for a final vote.

The standard for approval of each of the documents presented below was endorsement by a majority of the boards of CNS member organizations. This was necessarily a deliberate and time-consuming process. For example, in took over 2 years from the time of the original request until the taxonomy and entry level competency documents were approved by the full CNS. Following CNS approval, all of these documents have been submitted to CoS and posted on Clinical Neuropsychology’s page on the COS website (www.cospp.org/clinical-neuropsychology). Note that the intended audiences for these documents are overlapping but varied. For example, the taxonomy is intended to support standard language used by training directors at different levels of training. The goal is transparency, so that prospective trainees can have clear understanding as to what programs offer. The competency document is also of clear relevance to training directors and trainees but is also meant to inform credentialing organizations. The target audience for the post-doctoral competency document was specifically the APA Commission on Accreditation (CoA), which is evaluating its methods for accreditation at the post-doctoral level. Despite the differing audiences all documents are pertinent to education, training, and credentialing in clinical neuropsychology. The members of CNS felt these three documents should be presented together in this report. The goal of this report is increase awareness of these documents, enhance understanding of their purpose, similarities, differences, and make them more accessible and citable.

## Taxonomy for Education and Training in Clinical Neuropsychology

Through the process described above a taxonomy for clinical neuropsychology was developed by CNS and approved by its members. It is presented in Table 2.

**Table 2.** Taxonomy for education and training in clinical neuropsychology

	Doctoral^a^	Internship^a^	Postdoctoral^a^	Post-licensure^a^
Major Area of Study	Minimum of 1) Three neuropsychology^b^ courses, 2) two clinical neuropsychology practica^c^, 3) additional coursework, practica, or didactics in clinical neuropsychology^d^, AND 4) dissertation or research project in neuropsychology	1) At least 50% of training time in clinical neuropsychology AND 2) didactic experiences consistent with Houston Conference guidelines for knowledge^e^ and skill^f^.	11) Two-years full-time (or the equivalent) of formal training in clinical neuropsychology, with relevant didactic, clinical, and research activities (including assessment and intervention that incorporate neuropsychological theories, perspectives, or methods and exposure to related healthcare disciplines).	N/A
Emphasis	1) Two neuropsychology courses^b^ AND 2) two clinical neuropsychology practica^c^	>30% and <50% of experience in clinical neuropsychology supervised by a clinical neuropsychologist.	N/A	N/A
Experience	1) One or two neuropsychology course(s)^b^ AND 2) one clinical neuropsychology practicum^c^	>10% and <30% of supervised experience in clinical neuropsychology	N/A	N/A
Exposure	1) One neuropsychology course^b^ OR 2) one clinical neuropsychology practicum^c^	5%–10% of supervised experience in clinical neuropsychology and/or didactic training.	N/A	Any hours of CE in clinical neuropsychology

*Note*: As per APA guidelines all supervision in clinical neuropsychology must be provided by persons with competencies in clinical neuropsychology, aka, a clinical neuropsychologist.

^a^At the doctoral and internship training levels, it is recognized that all programs must meet the broad and general requirements for accreditation by the American Psychological Association (APA) or the Canadian Psychological Association (CPA). At the postdoctoral training level, it is recognized that the Major Area of Study is consistent with training standards for specialty accreditation in clinical neuropsychology through the APA. Regarding all levels of training, guidelines for specialty education and training in clinical neuropsychology are specified in the Houston Conference Guidelines, Hannay et al. (1998).

^b^To be a neuropsychology course, the course content must prominently address areas outlined in the Houston Conference Guidelines policy statement, Section VI.3 and Section VI.4. Additionally, the number of courses listed above assumes that courses are 3 credit hours each, within a semester system. As such, the Major Area of Study would require a minimum of 9 semester credit hours or 13.5 quarter credit hours, the Emphasis would require a minimum of 6 semester credit hours or 9 quarter credit hours, and the Experience and the Exposure would require a minimum of 3 semester credit hours or 4.5 quarter credit hours.

^c^Defined by practicum experience for equivalent of one academic year (e.g. 9 months, in semester or quarter systems) consisting of supervised training for at least 8 hr per week, with at least 50% clinical contact with patients in the provision of neuropsychological services.

^d^Additional training experiences can also include, but are not limited to, research experiences, lab meetings, brown bags, lecture/colloquia series, grand rounds, etc. and should be consistent with the guidelines for specialty education and training that are specified in the Houston Conference policy statement.

^e^Knowledge base. Clinical neuropsychologists possess the following knowledge. This core knowledge may be acquired through multiple pathways, not limited to courses, and may come through other documentable didactic methods.1. Generic Psychology Core: A. Statistics and methodology B. Learning, cognition and perception C. Social psychology and personality D. Biological basis of behavior E. Life span development F. History. G. Cultural and individual differences and diversity 2. Generic Clinical Core: A. Psychopathology B. Psychometric theory C. Interview and assessment techniques D. Intervention techniques E. Professional ethics 3. Foundations for the study of brain–behavior relationships: A. Functional neuroanatomy B. Neurological and related disorders including their etiology, pathology, course and treatment C. Non-neurologic conditions affecting CNS functioning D. Neuroimaging and other neurodiagnostic techniques E. Neurochemistry of behavior (e.g., psychopharmacology) F. Neuropsychology of behavior 4. Foundations for the practice of clinical neuropsychology: A. Specialized neuropsychological assessment techniques B. Specialized Neuropsychological intervention techniques C. Research design and analysis in neuropsychology D. Professional issues and ethics in neuropsychology E. Practical implications of neuropsychological conditions.

^f^Skills. Clinical neuropsychologists possess the following generic clinical skills and skills in clinical neuropsychology. These core skills may be acquired through multiple pathways, not limited to courses, and may come through other documentable didactic methods. Domains of skills and examples are: 1. Assessment: Information gathering. History taking. Selection of tests and measures. Administration of tests and measures. Interpretation and diagnosis. Treatment planning. Report writing. Provision of feedback. Recognition of multicultural issues. 2. Treatment and Interventions: Identification of intervention targets. Specification of intervention needs. Formulation of an intervention plan. Implementation of the plan. Monitoring and adjustment to the plan as needed. Assessment of outcome. Recognition of multicultural issues. 3. Consultation (patients, families, medical colleagues, agencies, etc.): A. Effective basic communication (e.g. listening, explaining, negotiating) B. Determination and clarification of referral issues C. Education of referral sources regarding neuropsychological services (strengths and limitations) E. Communication of evaluation results and recommendations F. Education of patients and families regarding services and disorder(s) 4. Research: Selection of appropriate research topics. Review of relevant literature. Design of research. Execution of research. Monitoring of progress. Evaluation of outcome. Communication of results. 5. Teaching and Supervision: Methods of effective teaching. Plan and design of courses and curriculums. Use of effective educational technologies. Use of effective supervision methodologies (assessment, intervention, and research).

^g^The residency experience must occur on at least a half-time basis.

## Entry-level Competencies in Clinical Neuropsychology

The competency document developed by CNS and approved by its members included a (1) preamble and an (2) extensive set of tables (Tables 3–10) enumerating the foundational and functional specialty competencies. The two-part document (preamble and competency tables) was included in the clinical neuropsychology specialty renewal petition submitted to CRSPPP in December, 2016. It is presented below.

**Table 3.** Foundational competencies unique to clinical neuropsychology but common across functional domains

*Cluster*/Foundational Domain	Competency encompassed by domain
*Scientific Knowledge and Methods*	The clinical neuropsychologist: Demonstrates knowledge of the clinical and cognitive neurosciences, including neurology, neuroanatomy, neurobiology, neuropathology, brain development, and neurophysiology.
	Maintains currency with key scientific developments in fields related to practice.
	Demonstrates and applies knowledge of scientific and scholarly developments in clinical neuropsychology.
*Evidence-Based Practice*	Understands key signs and symptoms of disease processes relevant to practice and how patient characteristics (e.g., demographic factors, comorbidities) affect their expression.
	Understands age-related changes in brain functioning and behavior across the lifespan.
	Understands the scientific basis for assessment strategy, including test selection, use of appropriate normative standards, psychometric and operating characteristics, and test limitations.
	Understands patterns of incidence, prevalence (i.e., base-rate), and natural course of conditions of interest in neuropsychology
	Appreciates decision-making strategies and their applications in differential diagnosis.
	Knows the scientific basis for diagnostic conclusions across a range of neuropsychological disorders.
	Incorporates and uses outcome research in neuropsychology in guiding assessments and formulating interventions, integrating patient and contextual factors.
	Applies key components of evidence-based practice (i.e., best evidence, clinical expertise, and patient characteristics/culture/values) in selecting appropriate assessment and intervention approaches.
	Applies information technology to assess and evaluate best evidence to guide practice.
*Individual and Cultural Diversity*	Integrates knowledge of diversity issues in neuropsychological assessment, research, treatment, and consultation (e.g. health disparities, language differences, educational level, cultural context, literacy, individual differences).
	Understands and appreciates how cultural, linguistic, disability, and other demographic/socioeconomic factors affect the process and outcomes of neuropsychological assessments and the application of normative data and interpretations in specific populations.
*Ethical, Legal Standards and Policy*	Applies ethical concepts across a range of settings; demonstrates awareness of legal issues relevant to the professional activities of clinical neuropsychologists across settings, including healthcare, research, school, military/veteran, industry, and forensic (e.g., criminal, personal injury, disability determination, fitness for duty, etc.).
	Understands specific ethical and legal issues that are relevant to neuropsychologist’s activities across settings, including informed consent, third party assessments, use of technicians/psychometrists, third party observers, disclosure of neuropsychological test data, and test security.
*Professional Identity*	Demonstrates professional identity as a clinical neuropsychologist; understands the unique contributions of neuropsychology to different educational, healthcare, and forensic/legal contexts.
	Demonstrates awareness of the roles of clinical neuropsychologists, and how those roles vary across settings (e.g., practice, research, training, etc.) and assessment/intervention contexts.
*Reflective Practice/Self-Assessment/Self-Care*	Engages in reflective self-assessment regarding the dynamic knowledge base and skill sets necessary for practice in clinical neuropsychology across practice settings with the goal of improving skill level over time; understands limits of competence in particular populations or settings and seeks to lessen their impact through continuing education, peer supervision/consultation, or additional training as needed.
*Relationships*	Maintains effective and productive relationships with patients, families, caregivers, colleagues, team members, trainees/students, and communities across complex interprofessional settings.
	Communicates clearly and effectively through both oral and written means, integrating and explaining neuropsychological concepts and interpretations in a manner best suited to particular audience (e.g., other professionals, patients, families, and caregivers).
*Interdisciplinary Systems*	Demonstrates knowledge of key issues and concepts in related disciplines (e.g., neurology, psychiatry, neuroradiology, rehabilitation, education) the ability to communicate and interact knowledgeably with professionals across these disciplines.
	Understands the roles of other professionals with regard to patient care and integrates the perspectives of related disciplines into their case conceptualizations.
	Makes appropriate referrals to other health professionals as part of treatment planning.
	Is able to work as a member of interprofessional teams and collaborate with other professionals to contribute neuropsychological information to overall team diagnostic formulation, planning, and intervention.

**Table 4.** Functional competencies: assessment

Domain	Competency encompassed by domain
Knowledge-based competencies	The clinical neuropsychologist will have knowledge of: Neuropsychology of behavior, including information processing theories, cognitive/affective neuroscience, social neuroscience, cultural neuroscience, and behavioral neurology.
	Patterns of behavioral, cognitive, and emotional impairments associated with neurological and related diseases and conditions that affect brain structure and functioning.
	Neurochemistry, neuropsychopharmacology, neuroendocrinology, and related areas relevant to practice.
	Neurodiagnostic techniques relevant to practice.
	Effects of common systemic medical illnesses on brain functioning and behavior.
	Patterns of behavioral, cognitive, and emotional impairments associated with psychiatric disorders.
	Potential influences of motivational factors and assessment context on test performance.
	Medications used for common medical diseases and psychiatric disorders and their effects on brain functioning and behavior.
	Theories and methods of measurement and psychometrics relevant to cognitive abilities, social and emotional functioning, and brain–behavior relationships, including test development, reliability, reliable change, and validity approaches (e.g., construct, content, criterion, ecological).
	Potential functional implications of neuromedical conditions and neuropsychological impairments as they relate to everyday ability level, quality of life, and educational/working/social/living environments.
Applied competencies	The clinical neuropsychologist will be able to:
	Analyze and clarify referral questions based on the context, professional roles, and the patient/examinee presentation.
	Gather information key to addressing the referral question, including interview(s), targeted behavioral observations, and review of records.
	Appropriately select tests, measures, and other information sources consistent with best evidence and specific context of assessment, including assessment of performance and symptom validity, if relevant.
	Appropriately administer and score tests and measures.
	Interpret assessment results, with formation of an integrated conceptualization that draws from all relevant information sources (e.g., interview, test results, behavioral observations, records).
	Provide recommendations for management that are appropriate to the assessment context and consistent with evidence-based practices.
	Demonstrate written communication skills in the production of integrated neuropsychological assessment reports.
	Provide feedback, as relevant to the assessment context, to patients, families, or caregivers in a sensitive manner adapting to the needs of the specific audience.
	Address issues related to specific populations (e.g. cultural or linguistic differences, physical or mental disability, use of interpreters, educational level) appropriately by referring to other providers with specialized competence, obtaining consultation, and describing limitations in assessment interpretation.

**Table 5.** Functional competencies: intervention

Domain	Competency encompassed by domain
Knowledge-based competencies	The clinical neuropsychologist will have knowledge of: Evidenced-based intervention practices to address cognitive and behavioral problems present in different clinical populations.
	Theoretical and procedural bases of intervention methods appropriate to address disorders of language, attention, learning and memory, executive skills, problem solving, perceptual processing, sensorimotor functioning, and psychological/emotional adjustment.
	How complex neurobehavioral disorders (e.g., aphasia, anosognosia, neuropsychiatric illness) and sociocultural factors can affect the applicability of interventions.
	How to promote cognitive health with patients through activities such as physical and cognitive exercise, stress management, and sleep hygiene.
	Empirically supported interventions provided by psychologists and other mental and behavioral health professionals.
Applied competencies	The clinical neuropsychologist will be able to:
	Identify targets of interventions and specify intervention needs.
	Employ assessment and provision of feedback for therapeutic benefit.
	Identify potential barriers to intervention and adapt interventions to minimize such barriers.
	Develop and implement treatment plans that address neuropsychological deficits while accounting for patient preferences, individual differences, and social cultural context.
	Implement evidence-based interventions in neuropsychological disorders.
	Independently evaluate the effectiveness of interventions employing appropriate assessment and outcome measurement strategies.
	Demonstrate an awareness of ethical and legal ramifications of neuropsychological intervention strategies.

**Table 6:** Functional competencies: consultation

Domain	Competency encompassed by domain
Knowledge-based competencies	The clinical neuropsychologist will have knowledge of: Professional roles and expectations of a consulting clinical neuropsychologist specific to each setting.
	Relevant literatures on the roles of neuropsychologists in consultation settings.
	Appropriate and contextually sensitive methods of consultation.
Applied competencies	The clinical neuropsychologist will be able to:
	Determine and clarify referral issues.
	Educate referral sources regarding the utility and relevance of neuropsychological services.
	Communicate findings from consultation activities effectively and efficiently.
	Provide effective assessment feedback and articulate appropriate recommendations in language appropriate for the audience.
	Provide effective consultation services within common settings and contexts in clinical neuropsychology practice.
	Communicate scientific findings within clinical neuropsychology in a manner that is relevant to the consultation setting and understandable to the recipient.
	Provide consultation in clinical research regarding brain–behavior relationships and appropriate neurobehavioral assessment strategies and tools.

**Table 7.** Functional competencies: research/evaluation

Domain	Competency encompassed by domain
Knowledge-based competencies	The clinical neuropsychologist will have knowledge of: The scientific method in generating neuropsychological knowledge and evaluating findings related to neuropsychological techniques, brain–behavior relationships, assessment strategies, and interventions.
	Research design and analysis relevant to clinical neuropsychological science and practice.
	The wide array of factors that mediate and modulate behavior and their implications for neuropsychological and related research.
	Performs research in an ethical and responsible manner, adhering to established national and institutional guidelines.
Applied competencies	The clinical neuropsychologist will be able to:
	Select research topics and perform literature reviews effectively.
	Demonstrate skills in conceptualizing, implementing, and interpreting research design and statistical analysis.
	Perform research activities, monitoring of progress, and evaluation of outcomes accurately and effectively.
	Communicate research findings effectively.
	Apply research methods in evaluating effectiveness of professional activities in clinical neuropsychology.

**Table 8.** Functional competencies: teaching/supervision

Domain	Competency encompassed by domain
Knowledge-based competencies	The clinical neuropsychologist will have knowledge of: Supervision theories, methods, and practices in professional psychology and clinical neuropsychology.
	Developmental stages in training that may impact the acquisition of clinical neuropsychology knowledge and skills.
	Ethical issues and state requirements relevant to teaching and supervision
Applied competencies	The clinical neuropsychologist will be able to:
	Provide effective teaching activities, presenting materials in an organized manner that is appropriate to the needs of the audience.
	Provide effective training to psychology trainees in the foundations of assessment, psychometric theory, and the administration and scoring procedures for tests and measures employed in clinical neuropsychology practice.
	Provide effective training in developing and asserting professional identity and role as a clinical neuropsychologist.
	Provide effective training in neuropsychological interviewing, test interpretation, case conceptualization, and the development of recommendations.
	Provide effective training in treatment planning and the provision of feedback.
	Demonstrate sensitivity to individual and cultural differences in supervisory contexts.

**Table 9.** Functional competencies: management/administration

Domain	Competency encompassed by domain
Knowledge-based competencies	The clinical neuropsychologist will have knowledge of: Administrative structures of practice settings relevant to neuropsychology.
	Common administrative and business practices needed to address prevalent assessment and consultation issues in neuropsychology practice (e.g., referral patterns, coding, billing, documentation).
	Methods and procedures for outcome assessment, program evaluation, and research in neuropsychology.
Applied competencies	The clinical neuropsychologist will be able to:
	Function effectively within administrative systems, educating others about role of neuropsychology and supporting structures with the goal of improving access to needed services.
	Implement administrative structures to address needs in neuropsychology practice settings (e.g., quality improvement, access to care, funding).
	Train and supervise technicians/psychometrists and monitor their skills following regulatory, ethical and legal standards.

**Table 10.** Functional competencies: advocacy

Domain	Competency encompassed by domain
Knowledge-based competencies	The clinical neuropsychologist will have knowledge of: Regulatory and policy initiatives that can affect provision of neuropsychology services and access to care.
Applied competencies	The clinical neuropsychologist will be able to:
	Apply scientific knowledge and skills in neuropsychology to advocate for needs of individuals/groups across systems and to advocate for equity and access to quality care.
	Collaborate with psychologists and other professionals to advocate for the profession and the specialty of neuropsychology.
	Educate the public about the nature and value of neuropsychology in healthcare.

### Preamble

#### Scope

The present document represents an inter-organizational effort promoted and moderated by the Clinical Neuropsychology Synarchy to delineate entry-level competences for clinical neuropsychology. It is important to emphasize that enumeration of entry-level competencies does *not* alter Houston Conference Guidelines (HCG). The HCG continue to describe the *process* of specialty training in clinical neuropsychology. Rather, this document describes the expected *outcomes* from following the HCG. These outcomes are enumerated in terms of practicable and measurable competencies. The HCG specify that rigorous, extensive and cumulative training in clinical neuropsychology takes place at the doctoral, internship, and postdoctoral levels but allows for flexibility regarding the level at which different trainees may achieve knowledge and skills. Similarly this document presents entry-level competencies cognizant that no single level of training imparts all competencies and that individuals may acquire these competencies in a varied fashion.

#### Background

The specialty training guidelines for clinical neuropsychology delineated in the Houston Conference statement, (Hannay et al., 1998a) have served the field well for almost 20 years. They have served as a specific but flexible guide for ***how*** to train in the field. A survey conducted in 2010 by the Inter-organizational Steering Committee on Education and Training (ISET) showed that Houston Conference guidelines have been widely adopted by training programs. Furthermore, those receiving training consistent with the guidelines rated themselves as being well prepared for practice (Sweet, Perry, Ruff, Shear, & Guidotti Breting, 2012). As such, the ISET saw no need for a wholesale revision of training guidelines, but acknowledged that a broadening of the field and new technologies may prompt the need for updates.

While Houston Conference Guidelines have been invaluable in specifying training structure they were less explicit in describing training goals, i.e., ***what*** the training structures described in the Houston Conference Guidelines should deliver. In the time since those guidelines were developed there has been increasing emphasis on defining competencies for professional practice, including within medicine (Epstein & Hundert, 2002; Williams et al., 2010) and psychology (Health Service Psychology Education Collaborative, 2013; Kaslow, 2004; Kaslow, et al., 2004; Roberts, Borden, & Christiansen, 2005; Rodolfa et al., 2005). As such, it has become increasingly important to express professional activities in terms of *practice competencies*. Clinical neuropsychology has yet to delineate detailed competencies for entry-level practice. At the point of its fourth petition for specialty status it behooves clinical neuropsychology to do so.

Because Houston Conference guidelines specify that a two-year postdoctoral residency serves as the culminating prerequisite for entry into practice in the specialty, defining entry-level competencies de facto defines the competencies expected of trainees at the completion of the postdoctoral residency.

Enumeration of these entry-level competencies will have the following benefits:

- Serve as a helpful resource for training programs, especially programs seeking accreditation at the postdoctoral level. Common materials could also be developed that greatly streamline the process of initiating and maintaining accreditation.
- Enhance the process of specialty credentialing of clinical neuropsychologists.
- Provide a framework for more senior clinical neuropsychologists to consider continuing education opportunities.
- Serve to identify the unique knowledge, skills, and abilities of clinical neuropsychologists that will enhance broad advocacy efforts in a changing healthcare environment.

#### Process

An initial effort to develop entry-level competencies was made by Rey-Casserly, Roper, and Bauer (2012) in Professional Psychology: Research and Practice. Those competencies were reviewed in detail by a task force established by the Clinical Neuropsychology Synarchy (CNS) which included Glenn Smith, CNS Chair, Neil Pliskin, SCN President, Paula Shear, SCN Past-President, Celiane Rey-Casserly, past Chair of the APA Committee on Accreditation, and Brad Roper, Chair of the SCN Education Advisory Committee, resulting in several wording changes from the original article. This first revision of the document was forwarded to the all CNS members on 1/4/2015 inviting comment. Initial reactions to the competencies were discussed at the CNS meeting in Denver in February 2015. Organizations then provided feedback in earnest over the course of the ensuing year. These comments were coalesced by Dr. Roper and discussed at the CNS annual meeting in Boston in February of 2016. At that meeting a subcommittee was formed to finalize integration of member organizations contributions into the competency documents. A second revision was submitted to all member organizations in the spring of 2016 requesting that the organizations affirm the committee’s accommodation of their input. The final document was included along with our petition for continued recognition as a specialty area to the Commission for the Recognition of Specialties and Proficiencies in Professional Psychology (CRSPPP) in December 2016.

*Structure.* The competencies are organized following recent developments in the classification of competencies into seven *foundational* competencies that cross multiple areas of practice (Table 1), and six *functional* competencies pertaining to specific domains of practice (Tables 2–7). These specific competencies in clinical neuropsychology build on foundational and functional competencies attained in professional psychology doctoral training, in many case providing the describing the application of generic health service psychology competencies (Health Service Psychology Education Collaborative, 2013; Kaslow, 2004; Kaslow, et al., 2004; Roberts et al., 2005; Rodolfa et al., 2005) in the field of clinical neuropsychology. The functional competencies are organized into elements that are knowledge based and elements that are skill based. Clinical neuropsychologists will not employ or demonstrate all competencies equally over the course of their careers. Some neuropsychologists may focus on assessment to the exclusion of intervention. Some private practitioners will do little teaching. However at entry into the field it is expected that they will possess all competencies and be able to demonstrate a preponderance of the competency elements listed in the tables.

#### Measurement

Consistent with Houston Conference Guidelines (HCG), the entry level for practice begins after completion of an APA-approved doctoral training program, APA-approved internship and a two-year postdoctoral residency. Each level of training already incorporates its own forms of interval assessments that are relevant to the competencies described herein. These start with candidate evaluations leading to graduate school admission, evolves through course exams and grades, qualifying exams, dissertation defenses, practica, internship, and post-doc supervisors ratings and culminates via passing written, practice sample, and oral board examinations. However, the enumeration of competencies will undoubtedly spark interest in developing comprehensive, level spanning systems of measuring and tracking trainee progress. Although assessing competency is not part of the current effort, programs and/or organizations may find consensus-based entry-level competencies helpful in developing such systems for their own use.

## Competencies at the Post-Doctoral Residency Level

In December of 2016, CoA sent a request to CoS to direct a project to identify and operationalize the specialty-specific competency domains that will be required of postdoctoral residency programs seeking APA accreditation. In turn, the CoS requested that each specialty council provide to CoS by July 1, 2017, specialty-specific, essential competencies in 16 competency domains. These domains are based upon ABPP competencies and also include of APA-CoA competencies:

- Integration of science and practice (note: an APA-CoA required competency)
- Ethical and Legal Standards/Policy
- Individual and Cultural Diversity
- Professionalism (professional values attitudes and behavior)
- Reflective practice/self-assessment, self-care
- Scientific knowledge and methods
- Interdisciplinary Systems
- Relationships
- Evidence-based Practice
- Assessment
- Intervention Consultation
- Research/Evaluation
- Supervision
- Teaching
- Management/Administration
- Advocacy

The competency domains Integration of Science and Practice, Ethical and Legal Standards, and Individual and Cultural Diversity are required for all postdoctoral residencies regardless of specialty. Specialties with competencies in any of the other 12 domains, where asked to consider which of those domains should be required during postdoctoral residency training in that specialty.

Specialty councils were asked to provide domain elements, operationalized in a specialty-specific fashion that are observable and easily evaluated. CoA plans to use these competency statements to populate the self-study instructions for postdoctoral residency programs undergoing review for accreditation in a given specialty.

CNS again empaneled a group of key stakeholders. The workgroup was led by Brad L. Roper, Ph.D., ABPP-CN (Co-Chair, Memphis VA Medical Center), and Amy Heffelfinger, Ph.D., ABPP-CN (Co-Chair, Medical College of Wisconsin). These leaders created a workgroup composed of five training directors drawn from the 25 postdoctoral programs in clinical neuropsychology that were APA-accredited at the time. In selecting workgroup members, consideration was made regarding proportion of representation from VA-based programs, DoD-based programs, academic medical centers and programs with pediatric neuropsychology subspecialty training. In addition to the chairs the workgroup included Karin J.M. McCoy, Ph.D., ABPP-CN (South Texas Veterans Health Care System), Robert A. Seegmiller, Ph.D., ABPP-CN (San Antonio Military Medical Center), and Jessica L. Vassallo, Ph.D., ABPP-CN (James A. Haley Veterans Hospital).

The CoA requires that all postdoctoral programs train and evaluate residents as specified in the Standards of Accreditation (SoA) and Implementing Regulation (IR) C-9P “Profession-Wide Competencies:” This includes competencies in:

- Integration of Science and Practice
- Ethical and Legal Standards
- Individual and Cultural Diversity

In addition to meeting IR C-9P, specialties may include additional elements of the above competencies that are unique to their specialty. In constructing the competencies, the CoS requested that specialties select from the list below:

- Professionalism (professional values attitudes and behavior)
- Reflective practice/self-assessment, self-care
- Scientific knowledge and methods
- Interdisciplinary Systems
- Relationships
- Evidence-based Practice
- Assessment

The specialty specific competencies to be address by post-doctoral residencies are listed in Tables 11 and 12. At the time of drafting of this manuscript the nominated competences for post-doctoral training in clinical neuropsychology were still under review from the CoA.

**Table 11.** Competencies, standards of accreditation level, and number of elements

Competency	Level	Elements
Integration of Science and Practice	1	4
Ethical and Legal Standards/Policy	1	4
Individual and Cultural Diversity	1	3
Professional Identity & Relationships/Self-Reflective Practice	3	5
Interdisciplinary Systems/Consultation	3	2
Assessment	3	5
Intervention	3	3
Research	3	2
Teaching/Supervision/Mentoring	3	2
Management/Administration	3	2
**Total Elements**		**32**

**Table 12.** Competencies and elements

**Integration of Science and Practice (Level 1)** Maintain currency of knowledge and skills in clinical neuropsychology practice, using scientific literature, seminars, conferences, training sessions, and/or other evidence-based resources. Demonstrate and utilize knowledge in the following foundational areas, including: o the neuropsychology of behavior, including information processing theories, cognitive/affective neuroscience, behavioral neurology, and lifespan neuropsychology. o additional areas as relevant to practice, especially neuroanatomy, neural systems, brain development, and neuropathology. Demonstrate and utilize knowledge in the following key areas, including: o theories and methods of measurement and psychometrics relevant to brain–behavior relationships, cognitive abilities, social and emotional functioning, performance/symptom validity, test development, reliability, validity, and reliable change; o scientific basis of assessment, including test selection, use of appropriate normative standards, and test limitations; o patterns of behavioral, cognitive, and emotional impairments associated with neurological, psychiatric, and general medical diseases and conditions which affect brain structure and functioning; o patterns of incidence, prevalence (i.e., base-rate), natural course, and key signs/symptoms of disease processes for conditions of interest in neuropsychology; o the potential functional implications of neuromedical conditions, psychiatric conditions, and neuropsychological impairments as they relate to everyday ability level, quality of life, and educational/working/social/living environments. Apply key components of evidence-based practice (i.e., best evidence, clinical expertise, and patient characteristics/culture/values) in selecting appropriate assessment, intervention approaches, recommendations, and supervision methods, and when engaging in consultation with other disciplines.
**Ethical and Legal Standards/Policy (Level 1)**
Are knowledgeable of, and consistently act in accordance with, o the current version of the APA Ethical Principles of Psychologists and Code of Conduct; o relevant laws, statutes, regulations, rules, and policies governing the practice of clinical neuropsychology at the organizational, local, state, regional, and federal levels; o relevant professional standards and guidelines. Are conversant with ethical and legal issues relevant to psychologists and neuropsychologists’ activities across settings, including informed consent, provider roles and relationships with patients/examinees, third party assessments, use of technicians/psychometrists, third party observers, disclosure of neuropsychological test data, test security, and assessment of performance/symptom validity. Recognize ethical dilemmas as they arise, apply ethical decision-making processes to resolve dilemmas, and utilize professional and legal consultation as appropriate. Conduct self in an ethical manner in all professional activities.
**Individual and Cultural Diversity (Level 1)**
Demonstrate an understanding of how their own personal/cultural history, attitudes, and biases may affect how they understand and interact with people different from themselves. Integrate current theoretical and empirical knowledge of diversity issues in neuropsychological assessment, research, treatment, and consultation (e.g. health disparities, language differences, educational level, cultural context, literacy, individual differences); understand and appreciate how cultural, linguistic, disability, and other demographic/socioeconomic factors affect the process and outcomes of neuropsychological assessments and the application of normative data and interpretations in specific populations. Demonstrate the ability to integrate awareness and knowledge of individual and cultural differences in the conduct of professional roles (e.g., research, services, and other professional activities). This includes the ability to apply a framework for working effectively with areas of individual and cultural diversity not previously encountered over the course of their careers. Also included is the ability to work effectively with individuals whose group membership, demographic characteristics, or worldviews create conflict with their own.
**Professional Identity & Relationships/Self-Reflective Practice (Level 3)**
Possess knowledge of the varying roles of clinical neuropsychologists across settings (e.g., practice, research, training) and assessment/intervention contexts. Demonstrate professional behavior and comportment that reflects the values and attitudes of clinical neuropsychology. Maintain productive relationships with a variety of individuals and demonstrate effective interpersonal skills, including the ability to manage difficult communication well. Engage in reflective self-assessment regarding limits of competence (e.g., knowledge base and skill sets necessary for practice). Exhibit awareness of personal and professional problems and demonstrate positive coping strategies with personal and professional stressors and challenges.
**Interdisciplinary Systems/Consultation (Level 3)**
Understand the key issues, concepts, and roles in related disciplines (e.g., neurology, psychiatry, neuroradiology, rehabilitation, and education) and other health professions, communicate effectively with other professionals, make appropriate referrals to them, and integrate their perspectives into case conceptualizations. Function effectively in consulting roles across settings (e.g., clinical, legal, public policy, research), clarifying referral questions, applying knowledge appropriate to each setting, and communicating results to referral sources both verbally and in writing.
**Assessment (Level 3)**
In neuropsychological assessment, accurately discern and clarify assessment questions, including who will be the “consumers” of the assessment results, and how assessment results will be utilized. Effectively gather information essential to addressing assessment questions, utilizing o clinical interviews; o targeted behavioral observations; o records reviews; o selection, administration, and scoring of neuropsychological tests appropriate to specific assessment contexts. Interpret assessment results to produce integrated conceptualizations, accurate diagnostic classifications, and useful recommendations. Communicate both orally and in written reports the results and conclusions of assessments in an accurate, helpful, and understandable manner, sensitive to a range of audiences. Address issues related to specific patient populations by referring to providers with specialized competence when appropriate, obtaining consultation, utilizing appropriate normative data, and describing limitations in assessment interpretation.
**Intervention (Level 3)**
Understand evidenced-based intervention practices to address cognitive and behavioral problems present in different clinical populations. Understand how complex neurobehavioral disorders and sociocultural factors can affect the applicability of interventions. Employ assessment and provision of feedback for therapeutic benefit.
**Research (Level 3)**
Accurately and effectively perform neuropsychological research activities, monitor progress, evaluate outcome, and communicate research findings. Apply knowledge of existing neuropsychological literature and the scientific method to generate appropriate research questions and determine effective research design and appropriate analysis.
**Teaching/Supervision/Mentoring (Level 3)**
Demonstrate knowledge of teaching, supervision, and mentoring theories, methods, and practices relevant to clinical neuropsychology. Teach, supervise, and mentor related to clinical neuropsychology effectively and appropriately.
**Management/Administration (Level 3)**
Possess knowledge of common administrative and business practices in neuropsychology practice (e.g., referral patterns, coding, billing, documentation). Manage responsibility for key patient care tasks and contacts with effective documentation in a timely manner.

## Future Directions and Conclusion

### Assessing Competence

As noted in the entry-level competencies preamble, enumerating competencies for a field has limited utility unless these competencies can assessed to be present or absent in individual practitioners. Establishing competence is of course a key function of credentialing organizations such as the American Board of Clinical Neuropsychology or the American Board of Professional Neuropsychology. In fact, at the time of the vote to approve the entry-level competency document there was already tight alignment between the document and those competencies included in the board guidelines and procedures that undergird the examination process. However, the capacity to conduct interim assessments of competencies throughout the training sequence will enable more effective and personalized training. Groups such as the APPCN are currently developing tools and methods for such interim assessment of competency acquisition.

### Subspecialty Competency

Clinical neuropsychology has also led the specialties in consider how to incorporate subspecialty practice. In 2014 pediatric neuropsychology became the first subspecialty area with an existing specialty area to be formally recognized by ABPP. It goes without saying that his subspecialty area has both unique and common competencies relative to the larger specialty. However, to date competency documents do not incorporate, reflect, or distinguish subspecialty issues. There is surely a need for this development in the future.

### Evolution

The Houston Guidelines (Hannay et al., 1998) were published twenty years ago. In the opinion of many, these guidelines served the field very well. It seems appropriate that in this 20th anniversary year additional documents pertinent to education and training in clinical neuropsychology should appear. These documents were developed at different times for different purposes, for different audiences. These distinctions are summarized in Table 13. It is unlikely that these documents will persist without modification for 20 more years. Indeed, the pace of specification and clarification in training models in professional psychology is increasing at the same time that the breadth and depth of neuropsychologists’ professional activities are expanding. Proficiency with new technologies may increasingly be required. Challenges in assessing competencies may result in revisions of how they are specified. Subspecialty recognition may result in the need for distinctive training pathways and competencies. The need to iterate the documents included herein will be a great sign of the vitality of our profession.

**Table 13.** Target, purpose and audience for education and training documents in clinical neuropsychology

Document	Applies to	Intended audience (in priority order)	Intended function	Not intended to:
Houston conference guidelines (Hannay et al., 1998)	Trainees	Trainees Credentialing organizations Training directors	Describe training pathways for clinical neuropsychology	Specify individual competencies
Taxonomy	Graduate programs, internships, post-doctoral residencies,and continuing education programs	Training directors Prospective trainees	Standardize terminology across training programs, training levels and specialties	Define education and training standards within a specialty Define competence
Entry level competency document	Individual neuropsychologists	CRSPPP Credentialing organizations Training directors, neuropsychologists in training	Describe basic competence in clinical neuropsychology.	Define training pathway (See Houston Guideline for this)
Competency document for post-doctoral level training	Post-doctoral training programs	CoA Post-doc training directors (eventually)	Nominate competencies for use by CoA in accrediting post-doctoral training programs	Define full entry level competence
